# Health systems performance assessment in low-income countries: learning from international experiences

**DOI:** 10.1186/1744-8603-10-5

**Published:** 2014-02-13

**Authors:** Christine Kirunga Tashobya, Valéria Campos da Silveira, Freddie Ssengooba, Juliet Nabyonga-Orem, Jean Macq, Bart Criel

**Affiliations:** 1 Institute of Tropical Medicine Antwerp, Nationalestraat 155, Antwerp, B 2000, Belgium; 2 Ministry of Health, 6 Lourdel Road Nakasero, P.O Box 7272, Kampala, Uganda; 3 Makerere School of Public Health, Mulago Hill, P.O Box 7062, Kampala, Uganda; 4 WHO Regional Uganda for Africa, P.O Box 6, Brazaville, Congo; 5 Institute of Research Health and Society, Catholic University of Louvain, Promenade de l’Alma, 31 bte B1.41.03, Brussels B-12000, Belgium

**Keywords:** Health systems, Performance assessment framework, Low-income countries, Uganda

## Abstract

**Background:**

The study aimed at developing a set of attributes for a ‘good’ health system performance assessment (HSPA) framework from literature and experiences in different contexts and using the attributes for a structured approach to lesson learning for low-income countries (LICs).

**Methods:**

Literature review to identify relevant attributes for a HSPA framework; attribute validation for LICs in general, and for Uganda in particular, via a high-level Ugandan expert group; and, finally, review of a selection of existing HSPA frameworks using these attributes.

**Results:**

Literature review yielded six key attributes for a HSPA framework: an inclusive development process; its embedding in the health system’s conceptual model; its relation to the prevailing policy and organizational set-up and societal context; the presence of a concrete purpose, constitutive dimensions and indicators; an adequate institutional set-up; and, its capacity to provide mechanisms for eliciting change in the health system. The expert group contextualized these attributes and added one on the adaptability of the framework.

Lessons learnt from the review of a selection of HSPA frameworks using the attributes include: it is possible and beneficial to involve a range of stakeholders during the process of development of a framework; it is important to make HSPA frameworks explicit; policy context can be effectively reflected in the framework; there are marked differences between the structure and content of frameworks in high-income countries, and low- and middle-income countries; champions can contribute to put HSPA high on the agenda; and mechanisms for eliciting change in the health system should be developed alongside the framework.

**Conclusion:**

It is possible for LICs to learn from literature and the experience of HSPA in other contexts, including HICs. In this study a structured approach to lesson learning included the development of a list of attributes for a ‘good’ HSPA framework. The attributes thus derived can be utilized by LICs like Uganda seeking to develop/adjust their HSPA frameworks as guidelines or a check list, while taking due consideration of the specific context. The review of frameworks from varied contexts, highlighted varied experiences which provide lessons for LICs.

## Background

Over the last three decades, efforts have been made to develop performance assessment frameworks that take into consideration the peculiarities of health systems, including the various determinants of health, the consideration of health (by some) as a public good, and the multiplicity of stakeholders in health with different perspectives on health systems performance [1-3]. These frameworks have been developed largely in the context of high-income countries (HICs) [4-6].

Many LICs struggle with questions like the following: How can health system stakeholders determine if the health system is (not) performing as it should? How can the reasons for this be established? What tool(s) can help governments carry out their stewardship role? An appropriate health systems performance assessment framework can be such a tool, help answer these questions, and support evidence-based decision-making. Such tools are important in all circumstances but are particularly crucial in LICs given the markedly limited resources versus the huge needs.

Health system performance assessment (HSPA) frameworks are determined primarily by issues high on the agenda in a health system. The key issues in health systems in HICs tend to revolve around containing costs while maintaining high-quality services, in an environment of advancing technology and high expectations from the society [5,7,8]. In low-income countries (LICs), however, the priority is increasing geographical and package coverage of basic services in the face of marked needs and minimal funding [9-12]. These differences in health system agenda issues reflect distinct differences in socioeconomic development and demographic and epidemiological profiles [13,14].

A few HSPA frameworks have been developed in LICs in the recent past, but most of the research on HSPA has been carried out in HICs; experiences in LICs tend not to be explicitly documented, and few have been studied [15-18]. An example of a HSPA framework in a LIC is the Uganda District League Table (UDLT), which was introduced in 2003 to compare performance among districts and determine ‘good’ and ‘poor’ performers, and the reasons why. This was in the context of devolution, with the mandate for overall stewardship, resource mobilization and allocation at the national level, and the management for service delivery at the district level. The table includes a number of input, process, and output indicators, some of which are used in a composite index for ranking the districts from the ‘best’ to the ‘worst’ performer [19]. The UDLT has been in use now for 10 years, and though it has been noted to have achieved some of the intended objectives, a number of challenges have been noted [20,21].

Most of the experiences and research in HSPA have taken place in HICs. However given the widely differing contexts, it is not desirable for a LIC country like Uganda to just copy these experiences. This paper is part of broader research aimed at studying the Uganda District League Table with the purpose of updating/adjusting it to provide an appropriate HSPA framework for the district level in Uganda today. This specific study has two objectives: to develop a set of attributes for a ‘good’ HSPA framework from literature and experiences in HSPA in different contexts; and to utilize the attributes for a structured approach to learning lessons from international experiences in HSPA. The attributes and lessons learnt will subsequently be utilized for updating the district health system performance assessment in Uganda. Other LICs seeking to develop/improve their HSPA frameworks can adopt the approach for their contexts.

### Working definitions of key terms used in this paper

The current interest in health systems has again brought to the fore the lack of agreement about how to define some of the related concepts. If we want to study HSPA frameworks, we need to agree on what we mean by a ‘health system’ and ‘health system performance assessment’ as before we can determine measures, we need clarity about what we are measuring [1]. In this article, we adopt the definition of a health system as detailed by the Lalonde paper, highlighting the broad determinants of health [22] and incorporating the concept of health actions, goals, functions, and building blocks elaborated by the World Health Organization (WHO) [23-25].

We also take into consideration the context, including population values and principles as further developed by Van Olmen et al., [26]. This definition of the health system is broader than what the term ‘healthcare system’ encompasses. Health systems have been observed to be composed of interdependent elements, with non-linear and dynamic relationships; extensive networks and feedback loops and time lags between an action and its effect. This has been referred to as ‘dynamic complexity’ and systems with such characteristics as complex adaptive systems [27-30]. These characteristics of a health system are acknowledged in our definition.

Smith et al. [8] define ‘performance measurement’ as seeking to monitor, evaluate, and communicate the extent to which various aspects of the health system meet key objectives. Measurement and assessment are often used interchangeably in the literature; however, some authors argue that assessment is a broader concept than measurement and involves collection, review and use of information for a purpose [31]. Sicotte et al. [32] building on previous work by Parsons [33] and Quinn and Rohrbaugh [34]-, presented the concept of health system/healthcare organization performance as maintaining a dynamic equilibrium among the major dimensions of the system namely: goal orientation; interacting with the environment; production; and maintaining internal values and norms. A framework has been defined as ‘a basic structure underlying a system, concept…’ [35]. The working definition of ‘a health system performance assessment framework’ for this paper, building on these definitions, is ‘a conceptually structured way of measuring the efforts of a complex and dynamic entity; with multiple actors working in various dimensions; whose main purpose is the improvement of people’s health; the analysis of such findings; and the application of the results to decision-making’.

## Methods

The study was carried out in three stages. The first stage, a literature review, served to generate an initial list of attributes for the HSPA framework. The second stage involved the validation/contextualization of the attributes to LICs using a Uganda-based expert group (EG); and the third stage used the attributes to review a selection of current HSPA frameworks for the purpose of learning lessons for LICs seeking to develop/adjust frameworks.

### Literature review

**Figure 1:**
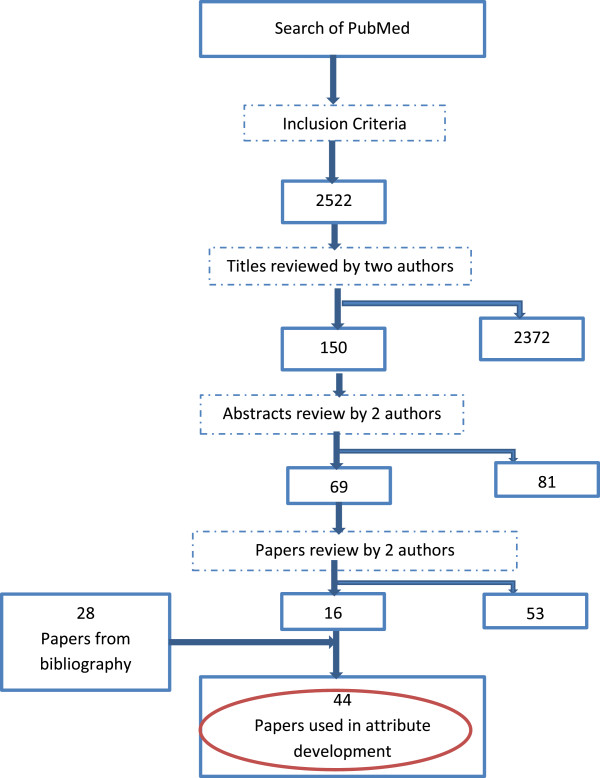
Approach to literature review for HSPA attributes development.

A structured review of the literature was undertaken to extract characteristics of a ‘good’ HSPA framework. The review began with a search of the PubMed database, using search terms ‘health system performance assessment’. Targeted articles were theoretical and empirical studies including reviews on HSPA that had been published between January 1995 and June 2013 in English. The initial search resulted in 2522 articles. A review of titles by the first author and one other author identified 150 relevant papers out of which 69 articles were selected after perusal of abstracts by the two authors. Consideration of entire papers yielded 16 relevant articles and a further 28 were identified through the bibliography. The final number of articles reviewed for the purpose of developing attributes for the HSPA framework was 44. Figure 1 illustrates this process.

### Expert group validation

The literature review yielded a number of characteristics that were grouped into six attributes (see Results) for a HSPA framework and discussed with the Uganda-based EG for validation for Uganda and LICs at large. The EG methodology involves the use of individuals knowledgeable in the topic and/or context of the study to provide focused input into the research process. Other researchers have used the EG approach in the development of clinical definitions, guidelines, and frameworks including HSPA frameworks [4,6,14,36]. The EG was intended to provide broader input into the research process; increase objectivity around the process and the validity of the findings for Uganda and other LICs, given the diverse specialties and organizations the EG represents; and improve uptake of findings of this study for decision-making in Uganda.

The EG meeting included 11 people purposively selected based on the following criteria: i) extensive experience (at least 10 years) in health systems including HSPA; ii) specialization in Public Health/Health Systems, Health Economics, Epidemiology, Statistics, Demography, Social Sciences, and/or Health Information Science; and iii) representation of a stakeholder organization including government (policy/service delivery level), donor organization, civil society organization, and academia/research. Table 1 shows the institutional base and specialization of the 11 participants. The EG held a one-day meeting; a week before the meeting, materials were circulated that included the research framework, attributes as developed from the literature review, and some key publications in HSPA. The first author and two rapporteurs were responsible for recording all proceedings. Key issues of concurrence and controversy were noted. The summary of the proceedings was discussed with participants and consensus reached on how to reflect the EG discussion.

**Table 1:** Expert group participants: affiliation and specialization

**Organization**	**Specialty**
Makerere University School of Public Health	Public Health/Health Systems Specialist (1)
Makerere University School of Public Health	Public Health/Monitoring & Evaluation (M&E) Specialist (1)
Private Consultant	Health Economist/M&E Specialist (1)
World Health Organization Country Office	Health Systems Specialist/PhD Student (1)
Ministry of Health/GFATM Coord. Office	Public Health/Health System Specialist (1)
World Health Organization Country Office	Health Systems/M&E Specialist (1)
CUAMM (NGO) Uganda Country Office	Health Systems/Hospital Performance Assessment (1)
PEPfAR Monitoring Unit Uganda	Public Health/Programme & District Assessment (1)
Uganda Catholic Medical Bureau	Health Systems/District & Hospital Assessment (1)
Uganda National Health Consumers Org.	Social Scientist/Health-care Consumers Advocacy (1)
Institute of Statistics, Makerere University	Statistician/PhD Student (1)

GFATM – Global Fund for AIDS, TB and Malaria.

PEPfAR President’s (US) Emergency Plan for AIDS Relief.

CUAMM Italian Non-Governmental Organization, Doctors with Africa.

### Application of attributes to HSPA frameworks

This aspect of the research involved applying the derived attributes to a number of current HSPA frameworks. The objective was to see how responsive the different frameworks were and in the process pick lessons for LICs seeking to develop/adjust their frameworks. HSPA frameworks were sought from peer-reviewed journals and from national and agency websites. Criteria used for framework selection was in line with our definition of a health system performance assessment framework, and included: being system wide (not just one programme, dimension, or service provider); well developed and documented, currently in use; and explicitly applied in a health system (or systems) at least twice. The frameworks were sought from high-, middle-, and low-income countries to provide the opportunity to learn from a range of contexts. Six frameworks were selected, and various methods were used to access information about them, including from peer-reviewed journals; and international agency, and national government websites. Identified frameworks were then reviewed using the attributes validated by the EG.

## Results

### Attributes of HSPA frameworks derived from literature review

From the literature, several characteristics of a ‘good’ HSPA framework emerged and were grouped into six attributes covering: process of development; clarity of the health system conceptual model; relationship with the policy/organizational context and societal values; content of the framework including a conceptual model, dimensions, and indicators; institutional set-up for performance assessment; and mechanisms for eliciting change in the health system. Each of these attributes is further elaborated on below.

A number of authors propose that attention should be paid to the *process of development* (*and review*) of the framework. Such a process should be inclusive, with the participation of key stakeholders [3,7,16,37-41], and involve explicit use of evidence to indicate causal links [8]. Sicotte et al. [32] emphasized the different values and preferences of stakeholders regarding performance of the system and the need to involve them at this stage, while Braveman [42] indicated that involving the various stakeholders would increase the perceived transparency of the process and enable them to appreciate the purpose and feed into the content. Policy makers were a special category of those indicated as should be involved, especially the top leadership of health systems, given that they both make decisions for the entities they oversee and have stewardship responsibilities with influence or leverage in other sectors/systems [8,43].

The HSPA framework should be *embedded in an explicit health system with clarity of* the conceptual model of the health system it references, including the determinants of health, system goals, elements, and actors [32,44-46]. Smith et al., [47] emphasized the need to delineate the scope of the health system, for example differentiating between one with a narrow focus on a healthcare system and a health system that includes the non-healthcare determinants of health. This approach, it has been argued, would focus designers of a HSPA framework on what is important to the health system [39].

The framework should *relate to the policy and organizational set-up and societal context* in which it is expected to work. This attribute includes paying attention to the general mode of government and specifically to the organization of the health system. The latter includes consideration of intra-system and inter-system linkages and the different levels (international, national, subnational, provider, and community), along with consideration of harmonization for comparisons across different levels [2,44,48-50]. A number of authors highlight the importance of considering societal values and principles as they vary across societies, yet are crucial in determining system goals and trade-offs [26,51]. The state of governance and empowerment (of entire societies and/or sections of society) has also been indicated as important given that it often determines the relationship between values and explicit policies [18,52-54].

The HSPA framework itself should be *well developed with a conceptual model, a purpose, dimensions and sub-dimensions, and indicators*. The importance of having an explicit purpose for the framework has been highlighted by some authors, and covers monitoring, accountability and improving management, focusing on one or more of these [3,7,47,54-57]. The conceptual model of the HSPA should be based on the definition and conceptual model of the health system, and the dimensions and sub-dimensions should reflect the linkages between different functions and/or elements of the health system [2,48]. Various approaches for partitioning the health system have been developed and used including those by: Donabedian ([58] structure/inputs, process, and outcomes), Kaplan and Norton ([59] the Balanced Score Card, BSC), and Sicotte et al., [32] (adaptation, goal attainment, production, and maintaining culture and values).

Specific measures against these dimensions to reflect progress (or lack of it) towards stated objectives, referred to as ‘indicators', should be described. The indicators should be parsimonious, and determined by what is important in the health system (strategic, linked to national/institutional goals). They should be scientifically acceptable (reliable, valid) and feasible [45,49,57,60]. Some researchers have argued that indicators should be selected taking into consideration: attribution for performance at system and entity level; facilitation of elaboration of link between processes and outcomes; reflecting what can be controlled by decision-making; and should not encourage perverse incentives [15,61].

The framework should be supported by an *institutional set-up for performance assessment* with appropriate resources (technological, financial, and human) and networks [6,8,36,41,43]. Arah et al. [15] refer to this set-up, including champions and linkages to targeted users of the information like financiers and regulators, as the ‘performance environment’. A combination of public and private entities has been espoused by some researchers given the nature of HSPA as may be viewed as a public good, whereas a number of the other players may be in the private sector [3,54]. The government has been particularly indicated as having an important role as sector steward, with responsibilities for definition of the conceptual framework, designing data collection mechanisms, information governance, developing analytical devices and capacity, designing incentives and managing the political process [47]. The framework should be regularly and systematically applied to be useful and relevant. Mannion & Davies [62] argued that such a set up should be a (nationally) coordinated system with mandatory participation and local flexibility/autonomy to be most effective.

The framework should explicitly *provide mechanisms for eliciting change in the health system* – indicating how the measurement of performance is linked to changes in policy, management, and delivery of services by various levels and players in the health system [55]. The cycle of performance measurement and management has been said to incorporate conceptualization of the health system and the performance framework, measurement of performance, analysis of data collected, the action necessary to change the behavior, and back to conceptualization of the health system [48,63]. This attribute focuses on the analysis and management action taken as a result of performance measurement and includes the presentation of information (from data analysis) and considerations of the target audience.

Some authors advise on how best to analyze and present these data, including the need to have a complete model with narrative information and ordinal and ratio indicators and charts and graphs. The presentation should consider the various audiences including policy makers, researchers, managers and consumers of care [39,62]. The approaches to effect changes in the health system in view of performance that have been proposed are varied. Certification and other forms of professional regulation and various quality improvement initiatives are the least controversial [64,65]. Public disclosure of performance information has been an issue of debate for some time especially in United Kingdom and United States of America, with recent studies indicating increased use for decision-making of such information by health system stakeholders like financiers and providers of services. The evidence is more mixed when it comes to influencing health care consumers’ behavior. Issues of ethics have also been raised in regard to public disclosure of information [55,66-68].

The use of incentives including payment for performance schemes has seen a marked increase in the last decade in both HICs and low and middle income countries [69,70]. Conrad [69] indicates variation of incentives along nature (reward or punishment), target entity (individual/group; provider/consumer), type (financial/non-financial), magnitude, frequency and whether intrinsic or extrinsic. A number of researchers though caution against the use of incentives and advise to look out for unintended consequences [69,70], and some researchers emphasize learning and not punishing [56,71].

### Expert group input

The EG considered the attributes of a HSPA framework generated from the literature from the perspective of their validity and applicability for LICs broadly and Uganda specifically. The EG concurred with the attributes derived from the literature and expanded and contextualized them as shown below. Table 2 provides a summary of key aspects of the attributes from both the review of the literature and the EG.

**Table 2:** Attributes of HSPA frameworks from literature review and input by expert group

**Attribute**	**Characteristics**	
	**From literature review**	**By expert group**
Process of development	• Participation of various stakeholders to bring on board various perspectives, increase transparency, appreciation, and ownership	• Some categories of stakeholders indicated include public, communities, and funders
	• Use of data to explain causal links	• Data use said to make framework more believable and more likely to be used for decision-making
Relating with health system framework	• Embedded in an explicit health system with clarity of HS conceptual framework including determinants of health, goals, elements, and actors	• May require working with stakeholders to develop health system conceptual framework if not in place already
Relating with policy/organizational context, societal values and principles	• Relating to general model of government	• Derivation of health system performance assessment attributes in this paper should be recognized as a specific perspective and not as generic
	• Relating to organization of the health system, inter- and intra-linkages at different levels of the system	
	• Societal values and principles determine system goals and trade-offs	
	• Governance and empowerment influence relationship between values and explicit policies	• Health financing – levels & structure – sources, mechanisms as one of the issues to monitor
		• Governance related to levels of literacy
The elaboration of the framework	• Includes conceptual framework, purpose, dimensions, sub-dimensions, and indicators	• Highlighting linkages and accountability relationships to facilitate attribution
	• Dimensions and sub-dimensions should reflect linkages between different functions and elements of the system	
		• Indicators – may require some flexibility & dynamism to allow for learning and ownership
	• Choice of indicators determined by perceived importance, scientific soundness, and feasibility	
Institutional set-up	• With appropriate institutional set-up, with linkages to other entities, champions, & resources (infrastructural, financial, human) provision	• Information management system requirements should consider feasibility & costs versus benefits
	• Regular and systematic application	
		• Should be usable at lower levels for self-assessment
Mechanisms for change	• Linking measurement of performance with changes in policy & management	• Packaging of information should consider types and needs of users
	• Making comparisons across time, different levels, systems, and settings	
		To consider negative/unintended effects of incentives including on data quality and increasing inequity
	• Analysis and use of complementary information from various sources	
	• Incentives – financial accreditation recognition – name and shame	
Adaptability		• History of use over time and in different places and contexts

The EG concurred with the aspects highlighted under *process of development* of the HSPA framework and indicated that some of the actors to watch for are those from public organizations – political, technical, and administrative (at different levels) – and from communities, civil society organizations, and funding agencies, including donors, health service providers, and professional groups. Availability of evidence particularly linking various dimensions of the framework was said to make conclusions from results more believable and likely to feed into decision-making.

On the HSPA framework being *embedded in an explicit health system*, the EG noted that in some instances, there may not be clearly articulated health system conceptual models, goals, and determinants of health. The EG consensus was that in such a case, it would be necessary to first work with stakeholders to develop the health system conceptual model, goals, and perceived determinants of health before the development of the HSPA framework.

In regard to *the policy/organizational context, values, and principles*, the EG emphasized certain aspects to monitor. One was the political organization of the country and specifically its implications for the health system. Uganda, for example, practices a decentralized model of government, with implications for institutional responsibility for population health. Districts have responsibility not only for provision of healthcare services but also for delivery of non-healthcare determinants of health like access to safe water. The EG indicated that it is important to examine the roles of government (at different levels), donors, and the private sector in health.

Another aspect considered important is health financing, levels, and structure. Important characteristics to consider are how much is spent in the health system, what proportion is from public versus private sources, what proportion is indigenous, and what proportion comes from external sources like aid. Other important considerations are what proportion of household spending on health is pooled and how much households pay directly. These factors have marked implications for many aspects of the health system and its performance, and as such, any HSPA framework should take them into consideration.

Regarding values and principles, the EG considered at length the perspective for determining attributes of HSPA frameworks (and the implications of this) and the entity that would be responsible for handling/applying these attributes in the development of the framework.

*“There seems to be an assumption that the criterion as is being developed is generic. Whose perspective is it?”* (Health Economist & Researcher)

“*Who is determining which characteristics of a HSPA are good? Who is to apply this list of attributes? Is this not important?* (Health Systems Specialist & Civil Society Organisation Employee)

The EG agreed that in this research work and specifically in the development of attributes for a HSPA framework, the perspective would be recognized explicitly as that of the authors with the input of the EG. For the application of the attributes in the process of development of a HSPA framework, the perspective would vary. Given the responsibilities of government as a steward, it is expected that in many cases, government officials would take the lead in such a process, working with other stakeholders.

In reference to the attribute of a *well-developed HSPA framework with a conceptual model*, the EG emphasized the need to highlight health system linkages and accountability relationships. This feature was considered pertinent given the multiplicity of stakeholders with varying responsibilities working at different levels of the health system. The EG held the view that there would be a need to balance indicators (numbers, type) given their strategic importance, data availability, and usefulness for decision-making and the interest and ownership of key constituencies/stakeholders.

The EG noted the importance of the state of information systems, especially the health management information system, in regard to *the institutional set-up* for performance assessment. The EG indicated that consideration needs to be given to the data requirements and the cost of acquiring these data in relation to the benefits provided. In most LICs, including Uganda, health management information systems are poorly developed, resulting in poor-quality data. Substantial resources are required to bring them to optimal levels. This situation, however, needs to be placed in perspective, and explicit prioritization for investment in HSPA should be made in the context of resources available for the health system and the country (or other level of government) at large. In addition, the EG discussed the issue of who was intended to receive the information from the application of the framework, including possible use for self-assessment.

“*The performance of an entity … for example, a district … depends on the performance of the units under it. It would be desirable for a district health management team to be able to use the framework to assess its performance and the performance of the units below it, in addition to submitting information upwards*” (Health Systems Specialist/Faith-based Umbrella Body Employee).

The consensus of the EG was that the potential for self-assessment should be taken into consideration in development of a HSPA framework.

The EG found the attribute of the *HSPA framework providing mechanism(s) for eliciting change in the health system* of great interest. It was emphasized that the use of the resulting information for policy formulation and decision-making at the various levels of the health system should be the main reason for having the framework.

*“What is the contribution of these assessments towards decision-making?”* (Social Scientist/Consumer Organization Employee)

“*How does this actually relate to policy formulation? Many frameworks are rather silent on this – is it because they are usually developed by technical experts only?”*(Monitoring and Evaluation Specialist/Researcher)

To facilitate use of the resulting information for decision-making, the EG emphasized the need for appropriate analysis of data including use of complementary sources of information and reference points, and the importance of packaging the information (data aggregation, presentation). It was noted that this process should be approached in such a way that the information produced would be accessible/used by different interest groups including technocrats, policy makers, and those with minimal literate and numerate skills. The EG considered the use of incentives (financial, recognition) but cautioned on unintended and undesirable effects, especially in circumstances of poorly developed/weak information management and oversight systems.

The EG introduced an additional attribute regarding *the adaptability of the HSPA framework to different contexts.* It was proposed that a history of use and or adaptation of the framework in different contexts (other than where it was developed), including LICs, would be an indication of its adaptability. Other aspects to look out for included the length of time the framework had been in use and changes made to improve or adjust the framework in view of major reforms in the health system or elsewhere.

### Application of attributes

Six HSPA frameworks were reviewed using these attributes to determine their responsiveness and for the purpose of extracting lessons for LICs intending to develop or review their frameworks. The frameworks were from/by: Australia, Canada, Ghana, the Netherlands, South Africa, and the WHO. Table 3 presents a summary of the characteristics of the HSPA frameworks, and the section below provides a brief analysis of experiences relative to each attribute.

**Table 3:** Highlights of selected health systems performance assessment frameworks

**Performance assessment framework**	**Attribute**						
	**Process of development & review**	**Health system framework**	**Policy, organizational, & societal context**	**Content of framework**	**Institutional set-up**	**Mechanism for change**	**Adaptability**
Australia National Health Performance Framework NHPF	• Work on PAF since the 90s	• The Lalonde model, appreciating both the healthcare & non-healthcare determinants of health	• Healthcare intended to be universally accessible	• Purpose: provide structure for reporting at national level & for developing PI sets for lower levels	• Rationalized and converged previous efforts at PA including indicator definitions, data processes, and local needs	• Present information in performance reports and HCAs	• Adapted from CHIRII
			• Shared responsibility by federal & state governments for funding, regulation, & provision of services				
				• Dimensions (2^nd^ edition of NHPF): Effectiveness, responsiveness, accessibility, safety, continuity, efficiency, & sustainability			
	• Led by national & state ministers & using technical experts						
		• Dimensions: health status & outcomes, determinants of health, HS performance				• National & international comparison	
			• Equity as key concern				
					• Linkage with generic national bodies responsible for funding & PA	• Accreditation & professionalism	
			• NHCAs outline goals & HS roles & responsibilities for government bodies	• Indicators emphasize: national standards, worthiness, relevancy, validity, reliability, priority (minority) groups, user understanding			
	• NHPF developed in 2001 & reviewed in 2009						
						• Accountability & consumer & participation	
							• Has been in use for more than 10 years – with review in 2009;
					• Involving a number of organizations: ACSQHC, COAG Reform Council, NHPAC, NHPC,, NICS, National HCAs		
						• Quality of care initiatives	
						• Epidemiological analysis linking inputs, processes, outputs, & outcomes	
							• Learning process with adjustment of dimensions, indicators, & reporting given current priorities, data availability, & possibility of interpretation
						• Financial incentives for building capacity for quality & safety	
Canadian Health Indicator Framework CHIF	• Initiated in 1998, endorsed by First Minister’s Meeting in 2000	• Lalonde model – appreciating healthcare and non-healthcare determinants of health	• Federal, provincial, & territorial levels roles & responsibilities	• To provide governments, providers, & public with reliable, comparable data across entities & assist in its use & interpretation	• Integrated network of HIS initiatives & structures, across country & levels including CIHI, SC, HC, CCHSA, CMA, AIM	• Biennial National Report	• Has been in use, evolving over more than a decade
			• Public (mainly) & private funding				
				• Domains: acceptability, accessibility, appropriateness, competence, continuity, effectiveness, efficiency, safety			
	• Built on previous work by CIHI and CCHSA						
				• Defined up to 70 indicators			
			• Various providers				
		• Dimensions: health status, non-medical determinants, HS performance, community, & HS characteristics				• Provincial & regional governments link to plans & targets	
							• Informed the development of frameworks for the OECD, Australia, & Netherlands
					• periodical pan-Canadian surveys for consumer opinion		
	• Wide consultation at national, regional and local levels;		• Minority populations with equity concerns				

	• Extensive use of evidence						
					• Marked financial & logistical investment over the last decade through CHIRII		
						• Benchmarking, CQI, Certification/Accreditation with professional bodies	

							• Change in indicators given data availability & interest
						• Accountability, through making Information available to public;	
						• Learning, innovation, sharing best practices	
					• National Consensus Conferences on Indicators		
Ghana Holistic Assessment of Health System	• Developed by the MoH and discussed with sector stakeholders, first time at the April 2009 Health Summit	• Health in center of national development agenda	• The assessment relates to the Health Sector PoW & the GPRS, guided by National Health Policy & MDGs	• Provide balanced and transparent assessment of sector performance indicating factors that may have influenced performance and suggest corrective measures	• Carried out by MoH & stakeholders & external reviewers	• Presented in briefs and reports discussed at national and regional forums	• Has been used for 4 years, to be adjusted with development of new PoW
					• Data mostly from HMIS, surveys, and KIIs		
		• Goals – child survival & RH, decreasing burden of disease, & health services availability & use					
						• Dashboard approach, with 3-step process: assessment of indicators & milestones, assessment against goals & targets, & assessment of whole sector	
					• Receives information from districts, regions, agencies, & MoH		
				• Uses 22 out of 34 PoW indicators			
		• Thematic areas: healthy lifestyle & environment, provision of health, RH and nutrition services, HS capacity development & governance & financing					
					• Marked challenges in data availability and quality – sanctions proposed for those who do not submit data as required		
						• Prizes proposed for good performers	
			• High donor contribution to sector including through the Multi-donor Budget Support, MDBS				
			• Decentralization, with geographical equity concerns				
Netherlands Dutch National Health System Performance Framework	• Consultative process between MoH & RIVM, & researchers over period 2002–2005	• Lalonde model for health determinants & Balanced Score Card (BSC) model of HSPA	• Transition from budget-driven healthcare system to regulated market	• Focus on technical healthcare quality, keeping other dimensions in sight	• Close working relationship between MoH & RIVM & researchers for ownership, & evidence base	• To provide evidence to make appropriate policy decisions	• Adapted from experiences in Canada (Lalonde model); and UK, US and Dutch healthcare organizations (BSC model)
						• Not really designed to link information with management strategy	
	• Used evidence in form of frameworks from elsewhere, consideration of roles of MoH & other stakeholders, & existing information infrastructure						
		• Interface of Lalonde model & BSC is the consumer, relating population health & health management	• Emphasis on transparency & results oriented management				
					• Linked existing databases; created new cost-effective sources of data as required		
				• BSC - consumer, financial, internal business processes & innovative perspectives			• Adapted in Ontario and & for OECD’s HCQI Project
			• BSC model adapted to a non-corporate, market-oriented entity				
				• Indicators selected in line with core questions posed on each perspective			
				• Compares healthcare performance with healthcare needs			
South Africa District Health Barometer SA DHB	• Developed by the Health Systems Trust (HST), a non-governmental organization in consultation with DoH	• Equitable access to good healthcare as a major goal of the health system	• Decentralized, with bulk of primary health care services funded by government	• To monitor progress & support improvement of equitable provision of PHC	• Housed by HST a private entity with research & HSPA skills, working in close consultation with DoH	• Annual reports with tables, graphs and maps comparing all districts and within metro and rural districts;	• Has been in place with annual publications since 2005
							• Adjustments made with improving data availability and quality and perceived needs for information
			• Post-apartheid inequality in access to healthcare			• Equity analysis,	
	• Research and consultation with experts						
	• Use of evidence						
						• Information to policy makers and managers at national, provincial & district levels &public domain including academic/research institutions	
				• Indicators: socioeconomic, input, process, output, outcome & impact, related to MDGs	• Uses secondary data from various government institutions		
			• Geographical equity a major issue				
					• Poor health information systems and quality of data cited		
				• For comparison of all provinces & districts and within the categories of rural and metropolitan districts;			
				• Equity as a major focus;			
				• Trends studied			
World Health Organization Health System Performance Assessment Framework	• Developed by WHO technocrats with wide stakeholder involvement only after the World Health Assembly of 2000 and marked criticism	• WHO introduced a number of concepts about a HS including health actions, boundaries, goals, functions and building blocks	• Intended as a l tool for use by all member states and therefore supposed to be generic and usable for assessment of and in widely varying contexts across the globe;	• For the purpose of helping member states to measure own performance, understand factors behind this and improve response;	• Global and national support for HSPA including establishment of EHSPI	• Presents information of member states in the World Health Report in league tables and plots;	• Has been in place since 2000 with substantial consultations following its launch; some adjustments have been made including dropping the composite goal performance index and elaboration of specific methodologies;
						• Utilise DALYs and DALEs as measures of overall population health;	
					• Development of tools and approaches for data collection and analysis		
	• Extensive use of evidence						
						• Computation of indicator of composite goal performance in 2000.	
		• Main (extrinsic) Goals indicated as: improving population health, responsiveness, & fair financial contribution					
				• Assessment of 5 components of the HS using a number of indicators: population health level and distribution; responsiveness level and distribution; distribution of financial burden;			
					• Use of WHO regional groupings, research institutions and international organizations for consultation;		
						• Relates DALES to health systems’ potential given country/health system resources.	• Has been adapted and used for subnational assessments and also adapted for use by Health Systems 20/20 in several countries.
						• Benchmarking and competition	
						• Public reporting & accountability	
				• Highlighting stewardship as important for system design, performance assessment, priority setting, inter-sectoral advocacy, rule setting, and consumer advocacy			

ACSQHC, Australian Council for Safety and Quality in Heath Care; AIM, Achieving Improvement Management; CCHSA, Canadian Council for Health Services Accreditation; CHIRII, Canadian Health Information Roadmap Initiative Indicators; CIHI, Canada Institute for Health Information; CMA, Canadian Medical Association; COAG, Council of Australian Governments; DALE, Disability-adjusted life expectancy; DALY, Disability-adjusted life years; DoH, Department of Health; EHSPI – Enhancing Health System Performance Initiative; GPRS, Ghana Poverty Reduction Strategy; HC, Health Canada; HCAs, Health Care Agreements; HCQI, Health Care Quality Indicators; HMIS, Health Management Information System; HS, Health System; HST, Health System Trust; KII, Key Informant Interview; MoH, Ministry of Health; NHCAs, National Health Care Agreements; NHPAC, National Health Priority Action Council; NHPC, National Health Performance Committee; NICS, National Institute of Clinical Studies; NQI, National Quality Institute; OECD, Organization for Economic Cooperation and Development; PAF, Performance Assessment Framework; PIs, Performance Indicators; PoW, Programme of Work; RIVM, (Dutch) National Institute for Public Health and the Environment; SC, Statistics Canada;[14,15,17,21-23,66-77].

Policy makers were involved, leading, or coordinating the process in the development of the HSPA frameworks, with the exception of the South Africa District Health Barometer (SA DHB), where the process was led by a private institution with HSPA expertise, the Health Systems Trust. Other stakeholders that often participated are experts in the areas of management, performance assessment, and health information systems; service providers; and funding agencies. Minimal involvement of service users was noted in these processes, documented only in Canada. The WHO, in the development of the framework in 2000, mostly used experts but opened the process to broader involvement of national level policy makers and researchers after widespread criticism. The use of data in the process of developing the frameworks was explicitly documented for Canada, Netherlands, WHO, and South Africa [9,14,15,24,72-75].

All of the frameworks indicate improving population health as a goal of the health system. There are variations, though, in terms of conceptualization of the health systems to which these frameworks relate. Australia, Canada, and Netherlands ascribe to the Lalonde model of broad determinants of health. The HSPA frameworks of Australia and the Netherlands then narrow down to measure performance within the healthcare system; at this point, the Netherlands framework uses the BSC model. The WHO framework was built around the health system as described in the World Health Report 2000, with a clear conceptual model and system goals [15,24]. There is less documented clarity regarding the health system conceptual models to which the Ghana and South Africa frameworks relate. The Ghanaian framework makes reference to health being at the center of national development; and the SA DHB, is said to relate to the Millennium Development Goals and the WHO HSPA framework [9,73]. The Canadian and Netherlands frameworks highlight the linkages between determinants of health and attempt to lay out accountability relationships among the different major actors in a health system [15]. The rest of the frameworks are not strong in this area.

The different frameworks have been developed (and revised) in specific policy/organizational contexts and in response to certain issues. For example, cost containment is an issue high on the agenda of most HICs; thus, the marked emphasis on efficiency in the frameworks of Australia, Canada, Netherlands, and WHO [15]. Equity is a principle that is common to all of the contexts, and as an example, geographical public health sector resources tracking is highlighted in the frameworks of Canada, Ghana, and South Africa, reflecting their particular circumstances. Decentralization as a form of government and market orientation of some of the healthcare organizations are reflected in the relevant frameworks [9,17,76,77]. Given the international mandate of WHO, the HSPA framework developed by the agency was intended to be generic, so as to be usable in different contexts [14,24].

The HSPA frameworks are elaborated to various extents, usually with a purpose, dimensions, sub-dimensions, and indicators, but the approach to partitioning the health system varied. The HICs of Australia, Canada, and the Netherlands have a similar approach and marked overlap in terms of dimensions [5,17,48,78]. The HSPA framework developed by WHO is the most detailed, with goals and functions of a health system and the related indicators [14,23,24]. The indicators vary, with a tendency to similar indicators at the higher (goal) level and variations at lower (processes, inputs) levels. Specific service/individual provider indicators are common in HICs, but population-based indicators are more common in LICs. The process and input level indicators tend to reflect the demography, epidemiology, and health financing structures in the different contexts [14,17,73,76,78,79].

The institutional set-up for performance assessment, including the information management systems, showed considerable variation. Canada was noted to have a well-developed consortium of public and private institutions that has benefited from a great deal of financial and technological investment over the last two decades [80]. Ghana and South Africa, on the other hand, are dealing with poorly developed and under-resourced systems with major gaps in quality of data [73,76,81,82]. The periodicity of application of country frameworks varies from monthly to quarterly, annually, and biennially.

All of the frameworks present performance data in reports, which are shared with key stakeholders, usually policy-makers, service providers, and funding agencies. The data are presented as-is and/or in comparison with other entities. Some frameworks attempt further manipulation of data. The Ghanaian version uses a color-coded dashboard arrangement to indicate good and poor performance at the national and regional levels. WHO ranks the performance of all member countries against the health system goals and publishes the rankings of countries biennially in the World Health Report. In 2000, WHO computed a composite index, an overall measure of system performance, and then compared this with the resources available to the country health system [21,67,68,75,83,84].

In the reviewed frameworks, benchmarking is expected to lead to improvements through internal mechanisms like accreditation and continuing medical education [47,84]. External mechanisms are also instituted to elicit change, including management approaches and financial incentives. Good performers are given incentives in the form of increasing autonomy and a chance to get additional funding and prizes (proposed in Ghana) whereas poor performers have to sign performance action plans (Australia, Ghana) [77,78]. Another external mechanism, sharing of information in the media and public forums, has been used in Canada and South Africa. In South Africa, putting the DHB information in the public domain is said to have led to use by policy makers at national and subnational levels for planning and resource allocation [79,85-87].

Some of the frameworks have been in place for more than a decade, namely those from Australia, Canada, and WHO. The Canadian framework informed development of the frameworks in Australia and the Netherlands [15,17,80,84]. The WHO framework is used to report on all member countries in the World Health Reports every 2 years and has also been adapted for use in a number of different contexts like the one-off subnational assessments in Uganda and Indonesia and those carried out by the Health Systems 20/20 in several countries [24,88,89]. The Canadian and WHO frameworks could therefore be said to be adaptable, given the length of time they have been operational and their use as the basis for the development of other frameworks. The Ghanaian and South African frameworks were developed much more recently, and their adaptability is as yet unknown [76,77].

## Discussion

There is a growing body of literature on HSPA, especially from the last three decades. The publishing of the World Health Report 2000 is one of the landmarks, and has stimulated debate, research and experimenting in HSPA. Most of the research and experiences are from HICs and international and regional agencies like the WHO and the Organization for Economic Cooperation and Development (OECD). In the more recent past, mostly since 2000, some low- and middle-income countries have developed frameworks and set up institutions for HSPA. However, there are few system-wide frameworks in LICs, and further still, research and publications on HSPAs in LICs are limited. Most of the material in this article on the Ghana and South Africa HSPAs has come from national/agency websites, with minimal information from peer-reviewed journals.

Some authors have proposed that rather than just copying models/approaches a structured approach to learning from experiences in health systems with differing contexts can be of benefit [90,91]. In HSPA it has been noted that with due recognition of cultural, economic, demographic, organizational and political differences, it is possible to learn from one another [17,62]. A structured approach has been taken in this research, whereby a combination of literature review for derivation of attributes of HSPA frameworks and validation of these attributes by a Ugandan-based EG were combined with the application of the attributes to review the experiences of six HSPA frameworks. This process has highlighted a range of experiences and provided an opportunity for learning lessons by LICs seeking to develop/adjust their HSPA frameworks. This section documents some of the lessons teased out by this exercise and a summary is provided in Table 4.

**Table 4:** Lessons and identified gaps from review of selected HSPA frameworks

**Attribute**	**Lesson**	**Identified gaps/Areas for further research**
Process of development	• It is possible and useful to involve a range of stakeholders in the development and review of HSPA framework as done in Canada, and involvement of researchers as in the Netherlands	• Limited involvement of beneficiaries of health systems
	• A private entity can act as lead agency as seen in South Africa	
Clarity of HS conceptual model	• Explicit HS conceptual models facilitate relating the HSPA framework to the HS model, e.g., the WHO HS model was developed just prior to developing the HSPA framework	• In the absence of explicit HS models, it is difficult to determine system goals and whether the right things are being measured
	• Explicit HS model coupled with clarity in partitioning the HS for PA highlights linkages and enables attribution – the Netherlands framework provides an example with the Lalonde HS model and the Balanced Score Card for the HSPA framework	
		• Lack of delineation between HS and healthcare systems provides challenges for HSPA
		• Contribution of healthcare to health often difficult to estimate, and responsibility for delivery and reporting on non-healthcare determinants is challenging
Relating to policy and organizational context	• Variations in context are reflected in the HSPA frameworks; 2 diverse examples provide different lessons for countries intending to develop HSPA frameworks:	• The effect of governance and various aspects of empowerment on HSPA and their relationship to literacy are not well documented
	○ Canada – very contextualized	
	○WHO – intended to support HSPA in member countries and thus fashioned generically	
Elaboration	• Similarities noted between HICs and differences between HICs and L/MICs at the level of dimensions and lower level indicators, with HICs emphasizing service and provider-specific indicators & L/MICs emphasizing population-based indicators	• There are still challenges in relating the different pieces of data in most frameworks to tell a story, to determine what is not working well
Institutional set-up	• Canada and WHO made substantial investment in HSPA including methodological aspects and technology for data collection, analysis, and dissemination, which has yielded results	• What is the right balance – how much do you invest in LICs given competing obligations?
	• Ghana & South Africa demonstrate that you can start simple & build useful systems for HSPA	
	• Champions for HSPA have been noted to have made an impact in Australia and Canada (ministers of health) and the Netherlands (researchers)	
Mechanism for eliciting change in the HS	• Working with various pieces of information from different sources validates, enriches, and supports interpretation for decision-making	• There is still limited information on what works in terms of eliciting change in the HS using HSPA, and more research needs to be done in specific contexts to learn more about this
	• Use of appropriate technology and strategies for analysis and dissemination helps provide information to more people, as seen in Canada	
		• There is not much noted in these experiences about unintended/negative consequences of HSPA
	• Combination of mechanisms (internal & external) facilitates change, as seen in Canada	
	• Combinations of stakeholder groups and skills (e.g., statisticians/researchers/policy makers/health managers/generic managers/professional bodies) facilitate decision-making – different combinations noted in Australia, Canada, Netherlands	
Adaptability	• HICs adapt HSPA frameworks from other HICs; LICs from international agencies	Given the contextual differences and their implications for HSPA, case studies of HSPA frameworks are likely to provide further understanding of what works (or does not) and why
	• The frameworks that have been in place for longer have evolved/changed with circumstances to remain relevant	

The review showed that the most common mode is to involve policy makers and technical experts at the national level in the development of the HSPA, with the government leading the process. Some countries/agencies, however, have shown that there are benefits from doing it differently. Canada’s extensive involvement of various stakeholders across several levels and constituencies, including civil society agencies, is seen as having contributed to a broader ownership of the framework [15]. In the Netherlands, the explicit involvement of researchers is said to have made the framework more evidence-based and has resulted in several publications on HSPA that provide information in the public domain about these experiences [75]. The development of the SA DHB by a private entity has shown that this approach is possible and has facilitated wide availability of the resulting information [87]. The appropriate mix of stakeholders will depend on the particular context. The limited participation of other stakeholders, including consumers of health system services, is a gap in many of the frameworks.

Different countries/agencies have different approaches to conceptualizing a health system. The elaboration of a health system conceptual model before the development of the HSPA framework by WHO, was ground breaking [14,23,24]. The use of the Lalonde model at the health system level and the BSC at the healthcare management level as in the Netherlands is interesting because it provides the possibility of highlighting accountability relationships and a way to introduce market orientation into the assessment [17]. On the whole, though, the contribution of healthcare to the broader health system and specifically improvements in health remains difficult to quantify [92]. The responsibility for assessment of and action on aspects of the health system beyond healthcare management is often ambiguous. This ambiguity is likely to be a bigger issue in LICs where major challenges in sanitation, education, social infrastructure, and access to safe water exist which are known to have a big impact on population health [53].

Reviewing the different HSPA frameworks in regard to the policy context of the various countries and values of the different societies revealed marked differences, as expected. A number of lessons can be learned by countries seeking to develop their frameworks, including the basic fact that contexts differ, which should be taken into consideration in the development of a HSPA framework. Some countries have taken particular efforts to make the frameworks relevant to their situations. The Canadian Health Indicator Framework (CHIF), provides a number of lessons: the consideration of the decentralized nature of the country; explicit consideration of minorities, including special efforts to collect data from these communities; and the emphasis on efficiency given cost containment as an issue in the Canadian health system agenda [15,74]. The WHO framework, on the other hand, sought to be generic because of the mandate of the organization and attempted to provide an approach that many countries could use. This however was the cause for controversy around the WHO HSPA Framework as it was deemed to imply universal values and/or their application [50,51,93,94].

Some of the differences between contexts, including between HICs and low- or middle-income countries and how they relate to HSPA frameworks, may be subtle although no less important. Examples include differences relating to levels of governance, literacy, various forms of empowerment, and expectations of the population. The involvement of civil society agencies in the development and use of the CHIF could be taken as an indication of the relatively well-developed state of governance in Canada whereas a private entity being responsible for the DHB may indicate a younger and possibly dynamic governance environment in South Africa [76,79].

The elaboration of the different HSPAs showed substantial similarities among the HICs and differences between them and the low- and middle-income countries. The HIC frameworks reflected a focus in performance assessment of the healthcare system and reflected the epidemiology, demography, and health financing arrangements in these countries. This focus was evident at the dimension, sub-dimension, and indicator levels, and especially with input and process indicators [5,15]. The frameworks from low- and middle-income countries’ on the other hand, maintained more population/health system level assessments in addition to reflecting health system organization, demography, and epidemiology. The preponderance of population level indicators may reflect health system agenda issues – the high morbidity and mortality levels, consideration of non-healthcare determinants of health, and concurrence with international aspirations like the Millennium Development Goals. However, it could also be the result of ambiguity in health system and HSPA conceptualization and challenges in data availability for the more specific service/provider level information [9,73]. Relating the different data pieces to create a story and explain inadequacies in performance was often not done in the different frameworks.

Experiences vary regarding the institutional set-up for HSPA. Possible lessons include that substantial and long-term investment in technology, methods, and networks is beneficial in building a robust HSPA set-up as in Canada and that champions are useful in putting and keeping HSPA on the agenda, as noted in Australia, Canada (ministers of health and heads of government) and the Netherlands (policy makers and researchers). However, some of the experiences show that it is possible to start modestly using routinely available data of less than stellar quality, as was done in Ghana and South Africa. Collaborations with other institutions/approaches have been useful in such cases as seen with the SA DHB and the South Africa Health Review [72,77]. Hyder AA (2003) proposed a similar approach for Pakistan [95]. This is likely to provide the stimulus needed to make the necessary investments in HSPA. Even the HICs frameworks considered in this study did not have all the required resources in place when they started, and substantial investment had to be made along the way [14,74]. It is important, though, to monitor for data availability and quality and have pragmatic plans for scaling up, including setting up systems for data quality assessment. A key question to ponder particularly in LICs is how much investment should be made in HSPA, relatively, given the many needs in the health system and beyond?

HSPA frameworks are useful if they affect decision-making, and experiences with the frameworks show that countries studied differ in this regard. Possible lessons include use of complementary data sources and analytical approaches; mixing internal and external mechanisms for eliciting change; and encouraging collaboration among different entities in the health system and beyond. The Canadian experience using a consortium including the Ministry of Health, Statistics Canada, and professional bodies like the Canadian Medical Association and Canadian Council for Health Services Accreditation, the use of technology and innovation, and provision of information to the public is of interest to note. The mechanisms employed for eliciting change are a combination of continuing quality improvement and public accountability [15,96]. In Australia, the emphasis is on a generic approach to management with the ministries of health at the different levels working together with management/accountability bodies to develop HCAs and financial incentives [15].

The above experiences show that it is important to think about how the framework will elicit change at the time of development and not later. Some of the frameworks though were not strong on this. Some authors argue that more effort is required in further analysis of the ‘black box’ between outcomes and activities from which they resulted. This is related to the nature of a health system as a complex adaptive system – multiple actors, non-linearity, unpredictability [27,29,30]. The use of evidence-based studies and consultation of stakeholders, and being explicit at what needs to be done, including the utilization of systematic outcome mapping – tracing of all the steps that lead to the outcome of interest - has been proposed. This has been favorably contrasted with just disseminating information and assuming it will lead to decision-making [71,97,98]. In the reviewed experiences, information was limited about the unintended and often negative effects of HSPA. Yet these have been documented as a major challenge by some authors from both the theoretical and empirical perspectives and are said to include tunnel vision, gaming, myopia, and misrepresentation of data [39,56,99].

A number of experiences with the development and implementation of HSPA frameworks was reviewed here. Some, like the CHIF and the WHO HSPA framework, have been in place for more than a decade; others, like the Ghana Holistic Assessment and South Africa District Health Barometer, are much more recent. Different processes have taken place in the different countries. It has been noted that countries are more likely to adapt frameworks from certain contexts, as shown by the example of the Netherlands and Australia adapting from the Canadian framework; Ghana and South Africa, however, were seen to have adapted from the generic example of the United Nations agencies, including WHO.

In addition to viewing the HSPA frameworks against individual attributes, it is important to see them as a whole. This perspective is necessary because it is good to have the comprehensive picture of the HSPA framework, and not relate with it in a piecemeal manner which might happen with a focus on individual attributes. Indeed this is essential given our working definition of a HSPA framework, and the ‘dynamic complexity’ of health systems as argued by some authors [27,30,39]. The attributes are obviously interrelated; for example, the process of development is clearly related to how the framework relates to the context and in turn, this is linked to the purpose, dimensions, and the indicators. The attributes thus bear a complex (non-linear) and context specific relationship to one another, in terms of content, timing, and the stakeholders to be involved. The relationship is likely to be influenced by amongst other things, whether it is new development or review of a HSPA framework; whether there is an articulated health system conceptual framework; the level of governance; and the level of development of systems including information management systems. Viewed comprehensively, the Canadian and WHO HSPA frameworks, though different, were responsive to a number of attributes and provided the widest range of lessons for LICs.

### Study limitations

This study faced some limitations. HSPA is a very broad area, and in this study we utilized a number of methods to derive and apply a number of attributes of a HSPA framework. Given considerations of space in relating this information, the material was highly summarized especially in regard to details about the various characteristics of attributes. Another limitation arose from using internet searches to source grey literature on HSPA and the selected frameworks. Information on websites is sometimes adjusted or even removed. This method could also introduce bias as it depends on material in the public domain.

## Conclusions

LICs seeking to develop/adjust HSPA frameworks need not reinvent the wheel. A structured approach to learning from the experiences of other countries/agencies including HICs can be useful. In this study literature review and input from a group of Ugandan experts provided a set of attributes for a HSPA framework. The attributes covered process of development, clarity of the health system model, relationship with policy/organizational context and social values, elaboration/content, institutional set-up for performance assessment, mechanism for eliciting change in the health system, and adaptability.

The attributes have been used to learn lessons from six currently in use HSPA frameworks. A number of lessons were noted from the experiences of other countries and agencies, and include (a) the recognition that it is possible to involve a range of stakeholders during the process of development and review of a framework and such inclusion is beneficial to the process; (b) explicit health system and HSPA conceptual models facilitate clarity of relationships between different entities and supports attribution; (c) policy and organizational context can be appropriately reflected in the framework, making it more relevant and usable; (d) there are marked differences between the structure and content of frameworks in HICs and low- and middle-income countries; (e) champions can make a positive difference for HSPA; you can start small, but substantial and sustained investment facilitates optimal functionality of HSPA; and (f) mechanisms for eliciting change in the health system should be developed alongside the framework. Some gaps in HSPA have been identified including: limited participation of those who benefit from the health system in development and use of HSPA frameworks; ambiguity in regard to health system conceptual models in some frameworks; inadequate distinction between the health system and the healthcare system; and a limited understanding of what works in terms of eliciting change in the health system.

The attributes developed in this study can form the basis of guidelines for a country/entity like Uganda seeking to develop or review its HSPA framework. More research on HSPA development and application, especially in LICs, is required to build the evidence-base to enable learning from different experiences.

## Abbreviations

BSC: Balanced Score Card; CHIF: Canadian Health Indicator Framework; EG: Expert Group; HCAs: Health Care Agreements; HIC: High Income Countries; HSPA: Health Systems Performance Assessment; LICs: Low Income Countries; L/MICs: Low/Middle Income Countries; M&E: Monitoring and Evaluation; OECD: Organization for Economic Cooperation and Development; SA DHB: South Africa District Health Barometer; UDLT: Uganda District League Table; WHO: World Health Organization.

## Competing Interests

JNO works for WHO in the Regional Office for Africa. We declare no other conflict of interest.

## Authors’ contributions

CKT and BC conceptualized the paper; CKT and VCdS carried out the literature review; all authors reviewed successive drafts of the paper. All authors read and approved the final manuscript.

## Authors’ information

CKT qualifications include: MB Ch B; MA Demography; Msc Health Policy Planning and Financing; She is currently undertaking a Ph D at Institute of Tropical Medicine Antwerp and Catholic University of Louvain. She is employed by the Ministry of Health, Uganda where she has worked in the health sector in Uganda for the last 22 years mostly in health policy formulation, sector monitoring and supervision;

VCdS qualifications include: MD; MPH; She lectures at the Institute of Tropical Medicine Antwerp, Belgium; She has worked in the health sector in Brazil and various African countries.

FS qualifications include: MB Ch B; MPH; PH D. He is a lecturer and Makerere University College of Health Sciences’ School of Public Health and has carried out extensive research and publication in health systems in developing countries.

JNO qualifications include: M B Ch B; Msc Health Economics; She is currently undertaking a Ph D at Institute of Tropical Medicine Antwerp and Catholic University of Louvain. She works for the WHO Regional Office for Africa in the health system cluster.

JM qualifications include: MD, MPH, Ph D. He is a Professor at Catholic University of Louvain, Belgium.

BC qualifications include: MD, MPH, Ph D. he is a Professor at Institute of Tropical Medicine Antwerp.
